# Incubation Behaviour of a Captive Female Great Grey Owl (*Strix nebulosa*) for an Unsuccessful and a Successful Hatching

**DOI:** 10.3390/ani15213168

**Published:** 2025-10-31

**Authors:** Zbigniew Kwieciński, Heimo Mikkola

**Affiliations:** 1 Department of Avian Biology and Ecology, Faculty of Biology, Adam Mickiewicz University, Uniwersytetu Poznanskiego 6, 61-614 Poznań, Poland; 2 Department of Biology, Kuopio Campus, University of Eastern Finland, Koskikatu 9B31, 80100 Joensuu, Finland; heimomikkola@aol.com

**Keywords:** Great Grey Owl, *Strix nebulosa*, incubation, incubation attentiveness—time on and off eggs, egg-turning frequency, ambient temperature, nest success

## Abstract

**Simple Summary:**

Reproduction is a fundamental aspect of avian biology, with incubation representing a critical phase—especially when only one parent incubates the eggs. Among owls, incubation behaviour remains insufficiently studied, and for the Great Grey Owl (Strix nebulosa), available information is especially limited. This study provides a detailed characterization of a captive female Great Grey Owl’s incubation attentiveness (time on and, time off eggs), egg-turning frequency, and the influence of ambient temperature on this behaviour. This study compared results from an unsuccessful (2008) and a partially successful (2009) nesting event. Prolonged incubation is often associated with infertile clutches, which may indicate adaptive or maladaptive behavioural responses. By comparing incubation behaviours between these two events, this study seeks to advance understanding of the reproductive ecology of the Great Grey Owl and contribute to broader insights into incubation strategies among owls. There were no significant differences in incubation duration (number of days) or egg-turning frequency between these nesting events. Comparable data from wild or other captive Great Gray Owl nests are lacking. Our findings suggest that incubation in Great Grey Owls is instinctive, shaped by evolution and not significantly altered by captivity conditions.

**Abstract:**

Great Grey Owl incubation patterns; knowledge of its breeding behaviour is limited. We used video recordings of a captive nesting female at the Poznań Zoological Garden to quantify incubation attentiveness (time on and off eggs) and other behaviours including egg-turning frequency, and the influence of ambient temperature on these behaviours. We also compared these behaviours for an unsuccessful nest (2008) and a partially successful (2009) nest. There were no significant differences between years for incubation duration (number of days) or egg-turning frequency. The female spent more time on eggs for the unsuccessful nest despite the total number of incubation days remaining unchanged. Ambient temperature influenced incubation behaviour, with the female adjusting its attentiveness (time on and off eggs) suggesting active thermoregulation. Our findings indicate that incubation in Great Grey Owls is instinctive and not affected by being held captive.

## 1. Introduction

Various avian incubation strategies have evolved based on a trade-off between current reproductive investment and future parental condition [1,2,3,4]. One of the main benefits of increased incubation attentiveness—that is, the proportion of time spent incubating the eggs—is improved hatching success and the quality of the hatchlings [5,6,7]. Failure to maintain optimal conditions (keeping the egg temperature within a suitable range) may lead to developmental disturbances, hatching failure, or embryo mortality [5,7,8]. Since incubation is an energetically demanding process, parents must behave in a way that allows them to meet their own nutritional needs while simultaneously ensuring appropriate conditions for the developing embryos [9,10,11].

Therefore, incubation and off-nest behaviours may be influenced by various environmental and temporal factors. This is particularly important in species in which only one parent incubates the eggs, as is the case with the Great Grey Owl [11,12]. For instance, ambient temperature can affect incubation behaviour both directly—by acting on the incubating bird—and indirectly, by altering egg and nest temperatures and bird metabolism. The time of day may also influence incubation dynamics through changes in light intensity, predator activity, or ambient temperature. As a result, incubation patterns may vary over the course of the breeding season [8,13,14].

Owl incubation behaviour, as an aspect of their ecology, remains poorly understood, despite recent technological advancements that have significantly facilitated the monitoring of avian incubation behaviour [15,16,17,18]. Understanding, among other aspects, the dynamics of this process contributes to a deeper comprehension of the biological and ecological strategies that underpin species continuity in these animals [12,19].

To date, no studies have documented the incubation patterns of the Great Grey Owl, and existing knowledge of its breeding behaviour remains limited. Available information pertains primarily to the timing of breeding onset, the approximate duration of egg incubation, clutch size, and the length of the post-hatching parental care period [20,21,22,23,24,25,26,27,28,29,30,31,32,33,34].

Studies conducted on owls and other animal species kept in captivity—such as in zoological gardens or rehabilitation centers—allow for the collection of detailed data on various aspects of their ecology in a more efficient and controlled manner than observations carried out in the wild [35,36,37,38]. However, it should be emphasized that results obtained under such conditions are limited by the distinct environmental parameters of captivity. Therefore, they primarily serve as a supplement to knowledge acquired through field research conducted in the species’ natural habitats [39,40,41,42,43,44].

On the other hand, an increasing number of bird species are currently threatened due to intensive human activity. As a result, conservation efforts based on ex situ breeding—including in aviary and captive conditions—are gaining importance. This situation highlights the need to deepen our understanding of incubation processes in birds kept under such conditions and to develop the ability to apply this knowledge in active species conservation programs [45,46,47,48,49]. In this study, we provide a quantitative assessment of incubation attentiveness in the Great Grey Owl, which enabled us to reliably document and distinguish parental behaviours in this species. The aim of this study is to provide a detailed characterization of incubation behaviour in the Great Grey Owl by quantifying several aspects of nest attentiveness. Specifically, we measured the following parameters: incubation attentiveness (time on or off eggs), egg-turning frequency, and the influence of ambient temperature on incubation behaviour. These variables were analyzed in relation to two reproductive outcomes: nests containing infertile eggs and nests that resulted in successful hatching. Prolonged incubation is often associated with infertile clutches, potentially reflecting either adaptive or maladaptive behavioural responses. Comparing incubation behaviours between two nest outcomes, enhances our understanding of the reproductive ecology of the Great Grey Owl and to contribute to broader insights into incubation strategies among owl species.

## 2. Materials and Methods

### 2.1. Study Area

The study was conducted at the Poznań Zoological Garden in 2008 and 2009. In 1999, a 7-year-old female and a 10-year-old male were paired for breeding and transferred to an aviary, with a total area of 350 m^2^. The observed breeding pair hatched in the wild in Finland. The pair initiated breeding attempts in 2000; however, successful reproduction occurred only in 2007, when a single chick hatched. The aviary was located in a woodland environment and the pair were fed captive-bred house mice (*Mus musculus*) and water was provided daily ad libitum (the birds were not supplemented with micro- or macronutrients). The owls also captured (occasionally) small rodents in the aviary, including the bank vole (*Myodes glareolus*) and the yellow-necked mouse (*Apodemus flavicollis*), as confirmed by pellet analysis. An artificial nest, approximately 1.5 m in diameter, was placed in one corner of the aviary (under a roof) at a height of about 6 m and was shielded on two sides with wooden paneling. The nest was oriented toward the northeast. Each year prior to the breeding season (January), the nest was refurbished by the staff—old lining material was removed and replaced with fresh hay and branches with pine needles (sometimes also with oak leaves). During observations, staff interaction was limited to providing food and checking the breeding status. The entire incubation process of the female Great Gray Owl took place under normal zoo conditions, where the birds were exposed to visitors. However, this did not disturb their incubation behavior, as the owls were accustomed to human presence—the female remained calmly on the eggs and showed no signs of agitation. The aviary was not intentionally illuminated at night; however, it remained partially visible due to ambient light from nearby street lamps illuminating the zoo’s internal roads and pathways. Additional monitoring was conducted using the zoo’s surveillance cameras installed in the vicinity of the aviary, which enabled detection of external activity, including the appearance of predators. This system also allowed observation of the owls’ behavioral responses, typically involving defensive attacks directed toward the aviary mesh in the direction of predators such as red foxes (*Vulpes vulpes*) and pine martens (*Martes martes*).

### 2.2. Video Surveillance and Analysis

The behavior of the Great Gray Owl pair was recorded using a video camera (Samsung, model OV-913C-Made in China (Shenzhen)). The camera was installed at a distance of approximately 1 m from the nest (at one of its sides) at an angle of about 70°, recording color video footage. It was equipped with infrared LEDs (ES-104), which enabled continuous 24-h monitoring, including nocturnal observations [15,16,17,45,49]. The video signal was transmitted via cable to analog recorder and stored on a 500 GB hard drive in daily sequences. Recording operations were managed using an ES-104 capture card and the dedicated Power CCTV software (4.0) Both the camera and the recorder had constant power supply. The recorder was located in a separate room about 100 m away from the aviary. Technical specifications were as follows: camera type: Samsung CCTV camera, model OV-913C, color; frame rate: 25 frames per second (fps); sensor: 1/4 CMOS (Samsung OV913C, OmniVision OV9130); resolution: 380–420 TV lines (analog, PAL/NTSC); video output: composite 1.0 Vp-p, 75 Ω (yellow RCA); lens: fixed-focus 3.6 mm (approx. 70° field of view); sensitivity: ~1.0 lux (without IR); infrared: ES-104 IR LED module; power supply: 12 V DC, current draw ~100 mA; audio: none.

The entire incubation period—from the laying of the first egg to the hatching of the chicks—was recorded. In the 2008 season, the recording spanned from April 12 to May 20, and in the 2009 season, from April 13 to May 20. Video data were analyzed by reviewing the footage using AVS Media Player software (4.0), and systematically recording observed behaviours in numerical form (i.e., frequency and duration) [17,45]. All monitoring time was recorded in the standard time zone UTC + 1.

### 2.3. Incubation Attentiveness (Time on or off Eggs) and Egg-Turning

Incubation attentiveness was quantified as the total time the female spent in direct contact with the eggs during each recorded hour of video footage. For example: time on eggs—start: 05:00:00, end: 05:14:38; time off eggs—start: 05:14:38, end: 05:15:19; time on eggs—start: 05:15:19, end: 05:50:38; time off eggs—start: 05:50:38, end: 05:51:19, and so forth. This procedure was applied consistently across the entire incubation period [50,51,52].

Time off eggs was defined as the duration during which the incubated eggs were not in contact with the female’s body and were thus exposed to direct environmental conditions, including ambient temperature [1,2]. Egg-turning frequency was defined as the total number of egg turns observed during the entire incubation period, expressed as the mean number of turns per hour per egg [53]. Egg turning was quantified both when the eggs were directly visible in the video during turning and by observing characteristic head movements of the female associated with egg turning.

To precisely determine the breeding status, the eggs of the Great Grey Owl were numbered. Accordingly, each time the female laid a new egg, the nest was checked to mark the egg. A similar procedure was followed to verify the contents of eggs after they were crushed by the female. During these inspections, it was recorded whether the eggshells contained dead embryos or were empty and mostly dry (no data on eggshell thickness were available).

### 2.4. Number and Time of Nest Departures

The number and duration of nest departures were defined as periods during which neither the female nor any adult bird was present on the nest or in its immediate vicinity (i.e., within the nest level in the aviary setting, within 0.5 m). These nest recesses constitute a subset of the total time off eggs [52].

### 2.5. Relationship Between Ambient Temperature and Egg-Turning Frequency and Time off Eggs

The effect of ambient temperature on egg-turning frequency and time off eggs was also compared between the two breeding seasons. For this purpose, temperature data from the Institute of Meteorology and Water Management in Poznań—Ławica branch were used. Temperature measurements were taken at hourly intervals throughout the day and night. The mean ambient temperature was 12.13 °C in 2008 (Cl: 11.06–13.20, range: +2 °C, +25 °C, *n* = 39), and 12.67 °C in 2009 (Cl: 11.77–13.57, range: −1 °C,+25 °C, *n* = 38). The difference in ambient temperature between the breeding seasons was no statistically significant (*t*_75_ = −0.778, *p* = 0.439). Additionally, for further analysis, the average temperature for each hour across the entire observation period was calculated.

### 2.6. Frequency of Male Food Deliveries and Female Feeding During Incubation

Frequency of male food deliveries to the nest was defined as the total number of visits by the male delivering food to the nest during the observation period, expressed as the mean number of food deliveries per day. Frequency of female feedings by the male was defined as the number of food items accepted by the female from the male during the incubation period, expressed as the mean number of feeding events per day [22,23].

### 2.7. Statistical Analyses

To characterize the relationships between the measured variables of incubation behaviour, Pearson’s correlation coefficient and Student’s *t*-test were applied. Statistical analyses were conducted using the IBM SPSS Statistics 2021 PL software package (version 21), following recommended methods of statistical analysis [54]. Mean values are presented throughout the text along with their corresponding 95% confidence limits (CL).

## 3. Results

We analyzed 1.794 h of video footage documenting the incubation behaviour of a pair of Great Grey Owls. The total observation time was 917 h in 2008 and 877 h in 2009, recorded over 39 and 38 days, respectively (Table 1).

**Figure 1:**
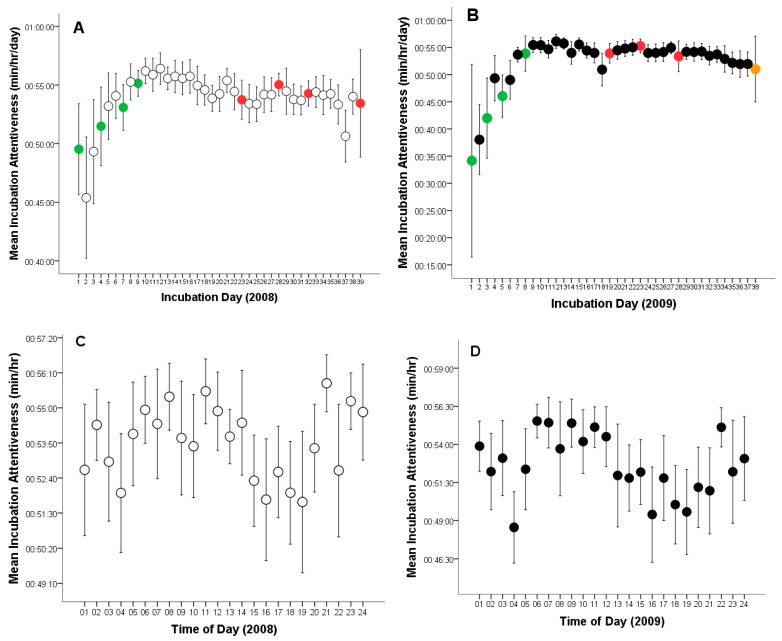
Variation in incubation attentiveness (time on or off eggs) by a captive female Great Grey Owl: (**A**) Incubation time of the 4-egg clutch of unhatched eggs during each monitoring day (white circles, green circles indicate the day of egg laying, red circles represent infertile eggs); (**B**) Incubation time of the clutch in which one eggs hatched during each monitoring day (black circles, green circles indicate the day of egg laying, red circles represent infertile eggs, orange circle marks the day of successful hatching); (**C**) Incubation time of the 4-egg clutch of unhatched eggs per hour (white circles); (**D**) Incubation time of the clutch in which one eggs hatched per hour (black circles). Mean values are given with 95% confidence limits.

On average, the female Great Grey Owl spent 87.8% of the time incubating eggs across both breeding seasons (Table 1). The overall mean incubation (time on eggs) was 20:40:38 h per day (CL: 19:53:46–21:27:31, range: 03:59:02–22:33:46, *n* = 77, Figure 1A, B). The mean time on eggs per hour was 00:53:15 min (CL: 00:52:56–00:53:32, range: 00:03:44–01:00:00, *n* = 1794, Figure 1C, D). On the other hand, mean time off eggs was 2:37:16 h per day (CL: 2:20:23–2:54:09, range: 00:39:20–08:47:29, *n* = 77), and 00:06:45 min per hour (CL: 00:06:27–00:07:02, range: 00:00:00–00:56:16, *n* = 1794).

Moreover, the total number of egg turns recorded across both breeding seasons was 2.872 times. The overall mean egg-turning was 37.30 times per day (Cl: 35.56–39.04, range: 9–55, *n* = 77). The average egg-turning frequency was 1.60 times per hour (Cl: 1.56–1.64, range: 0–5, *n* = 1794).

The total number of female departures from the nest was 275 times. The overall mean number of female departures from the nest was 3.57 times per day (Cl: 3.13–4.01, range: 0–12, *n* = 77), and 0.15 times per hour (CL: 0.14–0.17, range: 0–23, *n* = 1794).

The total duration of nest emptiness was 33:43:34 h (Table 1). The mean duration of nest emptiness was 00:26:17 min per day (CL: 00:17:59–00:34:34, range: 00:00:00–05:01:29, *n* = 77), and 0:01:06 min per hour (CL: 0:00:56–0:01:17, range: 00:00:00–01:00:00, *n* = 1794).

The male Great Grey Owl delivered food to the nest an average of 2.13 times per day (Cl: 1.64–2.55, range: 0–10, *n* = 77) (the total number: 165 times), while the female consumed food brought by the male an average of 0.88 times per day during both breeding seasons. (Cl: 0.66–1.10, range: 0–10, *n* = 77) (the total number: 68 times).

### 3.1. Breeding History

During the 2008 breeding season, the female Great Grey Owl laid her first egg on 12 April, during the early morning hours (01:00–02:00), marking the onset of incubation (Day 1) (Figure 1A). Subsequent eggs were laid at intervals of 2–4 days: the second egg on 16 April (17:00–18:00; Day 4), the third on 19 April (05:00–06:00; Day 7), and the fourth on 21 April (15:00–16:00; Day 9) (Figure 1A). All four eggs were infertile and were crushed during incubation, presumably due to the physical pressure of the incubating female. The crushing occurred as follows: first egg—4 May (after 23 days of incubation), second egg—9 May (after 24 days), third egg—14 May (after 25 days), and fourth egg—20 May (after 28 days and 6 h)(Figure 1A). After each incident, the female consumed the eggshells.

In the following season (2009), the clutch again consisted of four eggs. The first was laid on 13 April (17:00–18:00) (Figure 1B), followed by the second on 16 April (05:00–06:00; Day 3 of incubation), the third on 18 April (15:00–16:00; Day 5), and the fourth on 21 April (00:00–01:00; Day 8) (Figure 1B). The first three eggs were infertile and were likewise crushed during incubation. The female removed them by consuming the shells. The events occurred as follows: first egg—30 April (after 18 days of incubation), second egg—5 May (after 20 days), and third egg—10 May (after 23 days) (Figure 1B). In contrast to the previous year, the fourth egg was fertile. Based on 24-h nest monitoring, hatching occurred after 29 days and 6 h of incubation—on 20 May. The hatching process began on 19 May between 09:00 and 10:00, with the chick observed the next day (20 May) between 05:00 and 06:00 (Figure 1B).

### 3.2. Incubation Attentiveness (Time on or off Eggs), and Egg-Turning

In 2008, females spent an average of 21:06:02 h per day incubating the eggs (time on eggs) (CL: 20:12:44–21:59:20; range: 05:20:40–22:33:46; *n* = 39) and 02:24:43 h off the eggs (CL: 02:07:33–02:41:53; range: 00:39:20–05:50:46; *n* = 39, Figure 1A). The mean time on eggs per hour was 00:53:51 min (CL: 00:53:29–00:54:10; range: 00:05:05–01:00:00; *n* = 917), while the mean time off eggs per hour was 00:06:09 min (CL: 00:05:49–00:06:29; range: 00:00:37–01:00:00; *n* = 917).

In 2009, average hourly time on eggs was 20:14:35 h per day (CL: 18:55:06–21:34:04; range: 03:59:02–22:26:19; *n* = 38) and 02:24:43 h off the eggs (CL: 2:20:28–3:19:49; range: 00:45:00–08:47:29; *n* = 38, Figure 1B).

The mean time on eggs per hour was 00:52:37 min (CL: 0:52:08–0:53:07; range: 00:03:44–01:00:00; *n* = 877), while the mean time off eggs per hour was 00:07:23 min (CL: 00:06:52–0:07:51; range: 0:00:00–0:56:16; *n* = 877). In both years, females showed increased incubation activity in the early morning, around noon, and late in the evening (Figure 1C,D).

Time on eggs, measured in hours per day, did not differ statistically significantly between breeding seasons (*t*_75_ = 1.095; *p* = 0.277). Similarly, time off eggs was statistically insignificant (*t*_75_ = 1.512; *p* = 0.135).

However, incubation (time on eggs minutes per hour) was statistically significantly higher for the 4-egg clutch of unhatched eggs (2008, *t*_1792_ = 3.987, *p* = 0.0001; Figure 1), whereas time off eggs was significantly longer during the incubation period for the 4-egg clutch from which one chick hatched (2009, *t_1792_* = 4.077, *p* = 0.0001).

**Figure 2:**
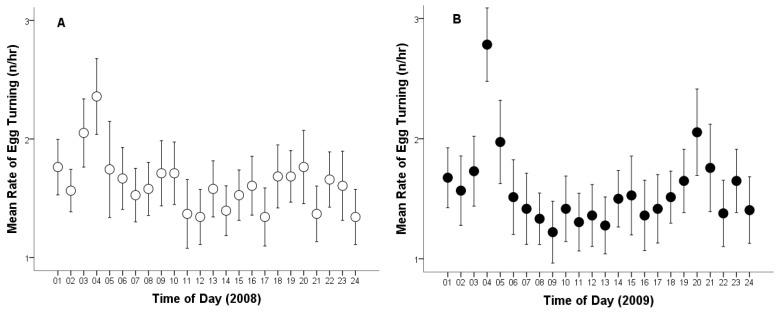
Average rate of egg turning per hour by a captive female Great Grey Owl during incubation: (**A**) in 2008 (4/4 eggs did not hatch); (**B**) in 2009 (3/4 did not hatch). Mean values are given with 95% confidence limits.

The mean egg-turning frequency per day in 2008 was 38.18 turns per egg (Cl: 35.94–40.42, range: 10–53, *n* = 39), and 36.39 in 2009 (Cl: 33.65–39.14; range: 9–55; *n* = 38). The mean egg-turning frequency per hour in 2008 was 1.62 turns per egg (CL: 1.57–1.68; range: 0–5; *n* = 917; Figure 2A), and 1.58 in 2009 (CL: 1.52–1.64; range: 0–5; *n* = 877; Figure 2B). No statistically significant difference was found in egg-turning frequency between the two breeding seasons (egg-turning frequency per day, *t*_75_ = 1.021; *p* = 0.310; egg-turning frequency per hour, *t*_1792_ = 1.121; *p* = 0.263; Figure 2).

### 3.3. Number and Time of Nest Departures

**Figure 3:**
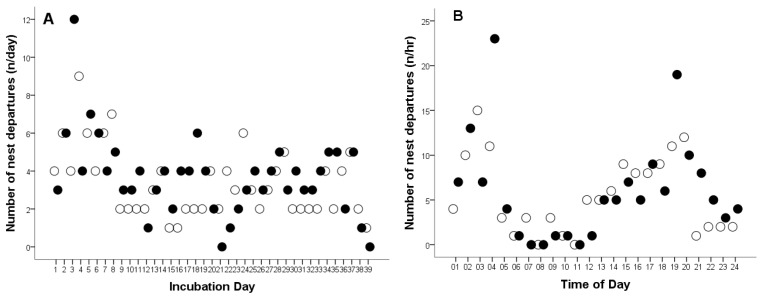
Number of nest departures by a captive female Great Grey Owl during: (**A**) the incubation day and (**B**) different times of day throughout the incubation period. White circles represent data from 2008 (4 of 4 eggs did not hatch), while black circles represent data from 2009 (3 of 4 eggs did not hatch).

In 2008, the total number of female departures from the nest was 131 times, a female Great Grey Owl left the nest an average of 3.36 times per day (CL: 2.7–3.95, range: 1–9 times, *n* = 39, Figure 3A), and 0.14 times per hour (CL: 0.12–0.17, range: 1–15 times, *n* = 917, Figure 3B). In 2009, the total number of female departures from the nest was 144 times. The average number of nest departures was 3.70 times per day (CL: 3.01–4.38, range: 0–12 times, *n* = 38, Figure 3A), and 0.16 times per hour (CL: 0.12–0.17, range: 1–23 times, *n* = 877, Figure 3B). No statistically significant differences were found in the frequency of nest departures by females during the incubation of infertile eggs compared to successful hatching (per day, *t*_75_ = −0.972, *p* = 0.334; per hour, *t*_1792_ = −1.254, *p* = 0.210).

In 2008, the average daily duration during which the nest was left unattended was 00:21:22 min (CL: 0:16:11–0:25:30, range: 00:04:06–01:05:28, *n* = 39, Table 1), and 0:00:54 per hour (CL: 0:00:43–0:01:05, range: 00:00:00–00:25:40, *n* = 917). In 2009, the average duration was 00:31:19 min pe day (CL: 0:14:57–0:47:41, range: 00:00:18–05:01:29, *n* = 38, Table 1), and 0:01:19 min per hour (CL: 0:01:00–0:01:39, range: 00:00:00–01:00:00, n = 877).

The difference in nest absence time during the incubation period between the 2008 and 2009 seasons was not statistically significant (per day: *t*_75_ = −1.199, *p* = 0.234), but was significant per hour (*t*_1792_ = −2.279, *p* = 0.023).

The outlier points shown in Figure 1C,D and Figure 3B, occurring between 03:00–04:00 and 19:00–20:00, represent the female’s responses (leaving the nest and interrupting incubation) and the male’s reactions to the presence of predators—red foxes (*Vulpes vulpes*) and pine martens (*Martes martes*)—near the aviary. These visits were likely attracted by the scent of uneaten prey remains within the owls’ enclosure. In 2008, eight such events were recorded, representing 6.11% of all nest departures (*n* = 131), whereas in 2009, 11 were observed, accounting for 7.64% of all cases (*n* = 144). These observations indicate that both adults displayed strong defensive behavior toward potential predators, although such disturbances had only a minor effect on the overall incubation pattern.

### 3.4. Relationship Between Ambient Temperature and Egg-Turning Frequency and Time off Eggs

**Figure 4:**
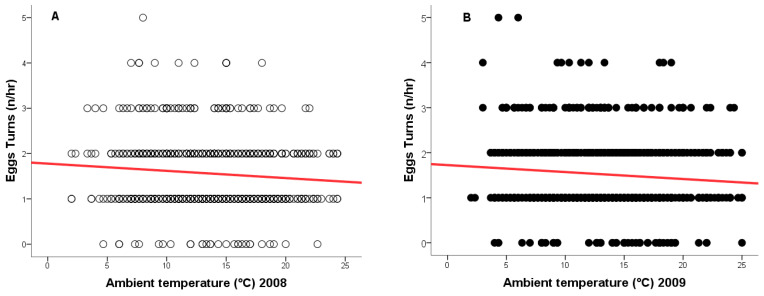
Relationship between ambient temperature (°C) and the number of egg turns by a female Great Grey Owl during incubation: (**A**) in 2008 (4/4 eggs did not hatch); (**B**) in 2009 (3/4 eggs did not 346 hatch).

**Figure 5:**
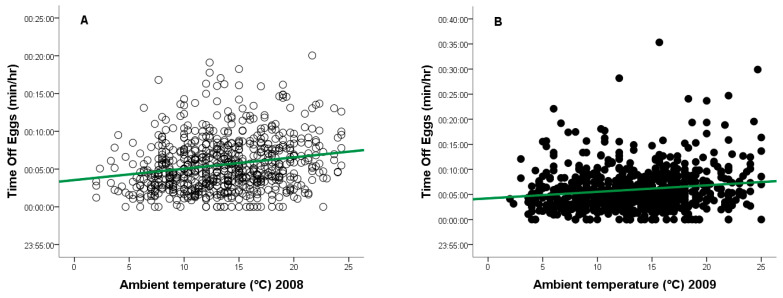
Relationship between ambient temperature (°C) and time off eggs for a captive female Great Grey Owl during incubation: (**A**) in 2008 (4/4 eggs did not hatch); (**B**) in 2009 (3/4 eggs did not 346 hatch).

During the 2008 breeding season, ambient temperatures ranged from +2 °C to +24 °C, and from −1 °C to +25 °C in 2009. As temperature increased, the frequency of egg turning decreased (2008: *r* = −0.148, *n* = 917, *p* = 0.0001, Figure 4A - red line; 2009: *r* = −0.118, *n* = 877, *p* = 0.0001, Figure 4B - red line), while the duration of time off eggs increased (2008: *r* = 0.133, *n* = 917, *p* = 0.0001, Figure 5A - green line; 2009: *r* = 0.124, *n* = 877, *p* = 0.0001, Figure 5B—green line).

### 3.5. Frequency of Male Food Deliveries and Female Feeding During Incubation

In the 2008 breeding season, a total of 107 male food deliveries to the nest were recorded. The mean number of deliveries per day was 2.74 deliveries (CL: 2.15–3.33, range: 0–10 times per day, *n* = 39).

During the incubation period, the female received food from the male on 50 occasions, with a daily average of 1.28 feedings (CL: 0.94–1.62, range: 0–4 times per day, *n* = 39). A statistically significant correlation was found between the number of male food deliveries to the nest and the number of feedings taken by the female (*r* = 0.701, *n* = 39, *p* < 0.0001).

In 2009, 58 male food deliveries to the nest were recorded, with a mean daily frequency of 1.53 times (CL: 1.00–2.05, range: 0–5 times per day, *n* = 38). The female took food from the male 18 times during incubation, with an average of 0.47 feedings per day (CL: 0.25–0.70, range: 0–3 times per day, *n* = 38). A statistically significant correlation was also found between the number of male food deliveries and the number of female feedings (*r* = 0.552, *n* = 38, *p* < 0.0001).

Significantly more male food visits were made (*t*_75_ = 3.112, *p* = 0.003) and significantly more food items were taken by the female in 2008 (*t*_75_ = 3.986, *p* = 0.0001).

## 4. Discussion

Incubation is a critical part of reproductive success in birds [1,2,9]. Increasing egg mass is generally associated with a prolonged incubation period and greater energetic investment required for reproduction, including foraging, territory or nest defense, and maintenance of body condition—particularly the female’s fat reserves [1,2,8,55].

The onset of incubation in the Great Gray Owl varied depending on the region and zoogeographical range of its occurrence [20,21,22,23,26,27,28,34]. The incubation period lasts 28–30 days, with females spending up to on eggs 98–99% of the time on eggs [1,21,22,23,26,27,28,31,34].

In Fennoscandia, the earliest clutches began in early April, although most pairs laid their first eggs in the second half of the month. However, substantial annual differences were observed, most likely linked to fluctuations in food availability. For instance, in one season the egg-laying period extended from 24 April to 10 May (mean: 1 May), while in the following season it occurred between 6 April and 28 April (mean: 18 April) [20,21,22,23].

In southeastern Idaho and northwestern Wyoming, the date of the first egg was significantly correlated with snow depth at the beginning of the breeding season. The mean laying date was 5 May, with the earliest eggs recorded on 19 April (under relatively shallow snow cover) and the latest on 23 May (under deeper snow conditions). In contrast, in northeastern Oregon females initiated clutches much earlier, between 17 March and 17 April. These findings indicate that snow depth is one of the key factors determining the timing of egg-laying and incubation [26,27,28].

Similar results were obtained during a 12-year observation of captive Great Gray Owls in Italy, where snow depth did not influence the onset of egg-laying or incubation. Under captive conditions, the first eggs were laid between 31 March and 25 April (mean: 12 April). This suggests that the phenology of reproduction in this species may be shaped not only by environmental and epigenetic factors but also, to some extent, by innate genetic mechanisms [56].

Our results under captive conditions support the suggestion that these behaviours are largely innate, with environmental factors in captivity exerting little to no influence on their expression.

Prolonged egg incubation is a rare phenomenon, although it has been documented in several bird species, including two owl species: the Barn Owl (*Tyto alba*) and the Long-eared Owl (*Asio otus*) [57,58,59]. It has been suggested that prolonged incubation may represent an adaptive mechanism providing a safety margin for chicks whose embryonic development takes longer than average [59,60,61].

One factor potentially prolonging incubation is the overall spread of hatching within a clutch [50,60]. Species with large clutches and asynchronous hatching may be more likely to extend the incubation period than species in which all chicks hatch within a short time window [58,59,62,63,64]. Furthermore, asynchronously hatching species may face greater difficulty in determining when to terminate incubation of unhatched eggs, owing to naturally higher intraspecific variation in the incubation period of viable eggs [50,59,60,64].

Prolonged incubation is also expected to occur more frequently in species where incubation is performed solely by one parent while the other (usually the male) provides food, compared to species in which incubation duties are shared. In the former case, the incubating bird does not face a trade-off between attending the eggs and foraging [11,12,55,62,63,64]. The mechanisms by which birds detect infertile or dead eggs remain unclear, as are the cues that trigger the cessation of incubation. Consequently, it remains unknown whether prolonged incubation is preceded or accompanied by other atypical behaviours that could act as proximate triggers [50,59,60,64].

We did not observe prolonged incubation in this study even though the most eggs were possibly infertile (no dead embryos were detected) and crushed before being incubated for 30 days [45]. During the incubation period, the female progressively crushed eggs under her own body weight. We consider this to be accidental and likely associated with eggshell fragility resulting from a nutritionally poor diet [5,6,36,42,45,50]. A low-diversity prey base may have led to deficiencies affecting the eggshell calcification process, resulting in thinner and mechanically weaker shells. As incubation progressed and eggs lost water, the shells became increasingly susceptible to mechanical failure, ultimately leading to their destruction [13,42,45].

The female studied spent more time on the eggs during the incubation period (measured in minutes per hour, but not in hours per day) [45]. Although comparable data for this species are lacking, the pattern we observed likely reflects a consistent incubation strategy rather than a specific response to egg infertility [1,2,50,51,59,60,64]. The slight statistical difference detected between seasons appears biologically negligible, suggesting stable parental behavior across years. Considering that the same female was monitored in both breeding seasons, this consistency further supports the interpretation that the observed incubation patterns represent individual behavioral stability rather than environmental or contextual variation. The length of the incubation period depends, among other factors, on the rate of embryo development within the egg [50,51,65], which is largely determined by active temperature regulation aimed at preventing overheating or cooling [7,8,13,65]. Optimal thermal conditions are maintained through time spent on or off eggs including shading of eggs, wetting them with water, and panting by adults during incubation or the influence of ambient temperature, observed throughout all stages of embryonic development [5,14,66].

Accurate quantification of egg thermoregulation is challenging, as some behaviours interpreted as resulting in cooling may result from stochastic events unrelated to parental activity, such as escape from the nest or predator pressure [13,50,51,52]. While time spent off eggs primarily serves to prevent overheating, little is known about egg tolerance to prolonged cooling, particularly in open-cup nests [50,52,65,66].

In this study, we found that time off eggs was significantly longer in the clutch in which only one of four eggs hatched. This may indicate that, in the Great Grey Owl, eggs containing a developing embryo require more time off egg, possibly due to ongoing physiological changes during embryonic growth. However, this interpretation should be approached with caution, as there is no conclusive evidence for a direct effect of time off eggs on hatching success [5,12,13,50,51,64,65,66]. The female we studied may have behaved in response to the prevailing environmental conditions, as evidenced by the demonstrated relationship between time off eggs and ambient temperature.

Another factor influencing proper embryo development is egg turning, which ensures even heating and cooling, facilitates gas exchange, and prevents the embryo from adhering to the eggshell throughout the entire incubation period [1,12,17,45,48].

Studies indicate that the number of egg turns depends on the length of the incubation period, the mass of the eggs laid, and, in particular, on the protein content [5,7,51,53]. Species in which the proportional content of protein—as a structural component of the egg—is high relative to other egg constituents tend to exhibit a relatively higher turning frequency. This is because protein undergoes the most rapid biochemical changes during incubation [50,51,53,60,67]. There are no published data on the proportional composition of egg fractions for this species [1,2].

We found no significant differences in egg-turning frequency between years. This may be due to similar outcomes for each clutch (4/4 unhatched eggs in 2008, and 3/4 unhatched eggs in 2009).

The influence of ambient temperature on egg-turning frequency and time off eggs is a well-documented phenomenon in many bird species. Weather conditions directly affect the behaviour of the incubating individual, triggering responses that enable the maintenance of optimal incubation conditions [8,11,51,53,66].

Previous studies have shown that environmental temperature affects not only the rate of embryo development, but also parental behaviours, including the frequency of egg turning and the time spent on eggs, which may serve as a compensatory strategy under thermally unfavorable conditions [11,65,66,67,68].

Field observation data suggest that, under variable climatic conditions, birds adjust both the rhythm of egg turning and the method of heating the eggs, compensating for fluctuations in external temperature. Under lower temperatures, they turn the eggs more frequently but spend less time off the eggs, which promotes even heating and minimizes the risk of thermal fluctuations within the egg [8,53,65,66,67,68,69,70].

This phenomenon has been described in other birds including gulls (Laridae), waders (Charadriiformes), and passerines (Passeriformes), where modifications of incubation behaviour allow the maintenance of stable embryo development conditions despite significant fluctuations in air temperature [71,72].

In our study, we observed similar patterns—an increase in ambient temperature resulted in less frequent egg turning by the female and a longer exposure of the eggs to atmospheric conditions. These results indicate that the female’s behaviour was innate and independent of the fertilization status of the eggs.

Our results are consistent with other reports that state that egg incubation is carried out exclusively by the female, whereas the male delivers food to the nest and feeds his partner [20,21,22,23,26,34,35]. Observations of a wild pair of Great Grey Owls in Finland showed that, during incubation, the female leaves the nest between one and five times per night, while the male delivers food to the nest three to four times per day [20,21,22,23,34]. This was consistent with the behaviour of the captive pair we studied.

The owls we studied under controlled, captive, and relatively safe conditions, reacted to appearances of potential predators (red fox and pine marten) near their aviary. In captivity they retained their ability to defend their “home range”, adjusting the intensity of their response according to the type and magnitude of the perceived threat.

Incubation behaviours appear to be innate and do not undergo significant weakening or spontaneous disappearance even after many years in captivity [45,73,74]. Studies on various aspects of the biology of birds kept in captivity indicate that their behaviours largely correspond to those observed in individuals living in the wild [45,73,74,75]. However, the lack of comparative data makes it impossible to determine the extent to which the incubation behaviours of the studied Great Grey Owls differ from those of individuals of this species under natural conditions.

A more recent video-based study on the incubation behaviour of the Eurasian Eagle Owl (*Bubo bubo*; hereafter Eagle Owl) was conducted at a nest in southern Germany [76]. The incubation period of the much larger Eagle Owl is longer (41 days), which naturally leads to some minor differences in egg turning frequency and nest absences. Male food deliveries caused the female to leave the eggs unattended when the prey was too large to swallow whole. Notably, large prey was never consumed at the nest during incubation. The Eagle Owl also breeds much earlier (beginning in February) than the Great Grey Owl, making it unfortunate that the author did not include weather data to assess the influence of cold temperatures on incubation.

Following these studies of the two largest owl species, numerous new webcam recordings have been made worldwide for various smaller owl species, both in captivity and under natural conditions. However, to date, no other detailed incubation studies on owls have been published. Analysis of breeding behaviour from such nest webcam recordings in other owl species should be strongly encouraged.

## 5. Conclusions

Our study provides the first detailed description of the incubation behaviour of a pair of captive Great Grey Owls with an unsuccessful clutch (2008) and a partially successful clutch. There were no significant behavioural differences between these cases in terms of incubation duration (number of days) or egg-turning frequency. However, time on eggs (measured as minutes per hour) was significantly higher when females incubated the unsuccessful clutch, even though the total incubation period was the same. It remains unclear whether prolonged time on eggs that do not hatch also occurs in wild populations, as comparable field data are lacking. We also found that ambient temperature influenced incubation behaviour, with females adjusting their time on eggs in response to changing weather conditions, suggesting active thermoregulation. Our findings indicate that incubation in Great Grey Owls is instinctive and not substantially altered by captivity conditions.

## Figures and Tables

**Table 1:** Nest attentiveness (time on or off eggs) and timing of nest departures by a female captive Great Grey Owl during the breeding period.

Behaviour	2008 Unsuccessful Clutch ^1^	2009 Partially Successful Clutch ^2^
Total Time of observation ^3^	917:00:00 (100%)	877:00:00 (100%)
Time on Eggs	822:55:42 (90%)	769:14:12 (86%)
Time off Eggs	94:04:18 (10%)	107:45:48 (14%)
In this:		
Nest Departure Time	13:53:19 (15%)	19:50:15 (18%)

^1^ Zero of four eggs hatched. ^2^ One of four eggs hatched. ^3^ Time in hh:mm:ss.

## Data Availability

The raw data supporting the conclusions of this article will be made available by the authors on request.

## References

[1] Deeming D.C. (2002). Avian Incubation: Behaviour, Environment, and Evolution.

[2] Reynolds S.J., Deeming D.C. (2015). Nests, Eggs, and Incubation.

[3] Wojczulanis-Jakubas K., Jakubas D., Kośmicka A. (2016). Body mass and physiological variables of incubating males and females in the European Storm Petrel (*Hydrobates p. pelagicus*). Wilson J. Ornithol..

[4] Birkhead T.R., Thompson J.E., Biggins J.D., Montgomerie R. (2019). The evolution of egg shape in birds: Selection during the incubation period. Ibis.

[5] Williams T.D., Groothuis T.G.G., Deeming D.C., Reynolds S.J. (2015). Egg quality, embryonic development, and post-hatching phenotype: An integrated perspective. Nests, Eggs, and Incubation.

[6] Birchard G.F., Deeming D.C., Deeming D.C., Reynolds S.J. (2015). Egg allometry: Influences of phylogeny and the altricial-precocial continuum. Nests, Eggs, and Incubation.

[7] Hepp G.R., DuRant S.E., Hopkins W.A., Deeming D.C., Reynolds S.J. (2015). Influence of incubation temperature on offspring phenotype and fitness in birds. Nests, Eggs, and Incubation.

[8] Conwey C.J., Martin T.E. (2000). Effects of ambient temperature on avian incubation behavior. Behav. Ecol..

[9] Nager R.G. (2006). The challenges of making eggs. Ardea.

[10] Mainwaring M.C., Hartley I.R. (2013). The energetic costs of nest building in birds. Avian Biol. Res..

[11] Nord A., Williams J.B., Deeming D.C., Reynolds S.J. (2015). The energetic costs of incubation. Nests, Eggs, and Incubation.

[12] Marasco V., Spencer K.A., Deeming D.C., Reynolds S.J. (2015). Improvements in our understanding of behaviour during incubation. Nests, Eggs, and Incubation.

[13] Grant G.S. (1982). Avian incubation: Egg temperature, nest humidity and behavioral thermoregulation in hot environment. Ornithol. Monogr..

[14] Ward D. (1990). Incubation temperatures and behavior of Crowned, Black-winged, and Lesser Black-winged Plovers. Auk.

[15] Cooper B.C., Mills H. (2005). New software for quantifying incubation behavior from time-series recordings. J. Field Ornithol..

[16] Bolton M., Butcher N., Sharpe F., Stevens D., Fisher G. (2007). Remote monitoring of nests using digital camera technology. J. Field Ornithol..

[17] Smith J.A., Cooper C.B., Reynolds S.J., Deeming D.C., Reynolds S.J. (2015). Advances in techniques to study incubation. Nests, Eggs, and Incubation.

[18] Fogarty T.D., Elmore R.D., Fuhlendorf D.S., Loss R.S. (2017). Influence of olfactory and visual cover on nest site selection and nest success for grassland-nesting birds. Ecol. Evol..

[19] Newton I. (1998). Population Limitation in Birds.

[20] Pulliainen E., Loisa K. (1977). Breeding biology and food of the Great Grey Owl, *Strix nebulosa*, in a north-eastern Finnish forest, Lapland. Aquilo Ser. Zool..

[21] Hilden O., Helo P. (1981). The Great Grey Owl *Strix nebulosa*—A bird of the northern taiga. Ornis Fenn..

[22] Mikkola H. (1981). Der Bartkauz Strix nebulosa.

[23] Mikkola H. (1983). Owls of Europe.

[24] Korpimäki E. (1986). Niche relationships and life-history tactics of the three sympatric *Strix owl* species in Finland. Ornis Scand..

[25] Bull L.E., Henjum G.M., Rohweder S.R. (1988). Home range and dispersal of Great Gray Owl in Northeastern Oregon. J. Raptor Res..

[26] Bull L.E., Henjum G.M., Rohweder S.R. (1988). Nestling and foraging habitat of Great Gray Owl. J. Raptor Res..

[27] Franklin A. (1988). Breeding biology of the Great Gray Owl in Southeastern Idaho and Northwestern Wyoming. Condor Ornithol. Appl..

[28] Bull L.E., Henjum G.M. (1990). Ecology of the Great Gray Owl.

[29] Mikkola H., Estafiev A.A., Kotchanov S.K., Hagemeijer E.J.M., Blair M.T. (1997). Great Grey Owl *Strix nebulosa*. The EBBC Atlas of European Breeding Birds: Their Distribution and Abundance.

[30] Sulkava S., Huhtala K. (1997). The Great Gray Owl (*Strix nebulosa*) in the changing forest environment of northern Europe. J. Raptor Res..

[31] Stefansson O., Mikusek R. (2005). Great Grey Owl *Strix nebulosa*. Methods of Research and Protection of Owls.

[32] Keller M., Chodkiewicz T., Woźniak B. (2011). Great Grey Owl *Strix nebulosa*—A new breeding species in Poland. Ornis Pol..

[33] Solheim R., Stefansson O. (2016). Life span, dispersal and age of nesting Great Grey Owls *Strix nebulosa lapponica* in Sweden. Ornis Svec..

[34] Bull E.L., Duncan R.J., Billerman S.M. (2020). Great Gray Owl (*Strix nebulosa*), version 1.0. Birds of the World.

[35] Cieślak M., Kwieciński Z. (2009). Moult and Breeding of Captive Northern Hawk Owls *Surnia ulula*. Ardea.

[36] Dickens M.J., Bentley G.E. (2014). Stress, captivity, and reproduction in a wild bird species. Horm Behav..

[37] Riyahi S., Björklund M., Mateos-Gonzalez F., Sena J.C. (2017). Personality and urbanization: Behavioural traits and DRD4 SNP830 polymorphisms in great tits in Barcelona city. J. Ethol..

[38] Kwieciński Z., Tryjanowski P., Zduniak P. (2024). Intersexual patterns of the digestive tract and body size are opposed in a large bird. Sci. Rep..

[39] O’Regan H.J., Kitchener A.C. (2005). The effects of captivity on the morphology of captive, domesticated and feral mammals. Mammal Rev..

[40] Kwieciński Z., Tryjanowski P. (2009). Differences in digestive efficiency of the white stork *Ciconia ciconia* under experimental conditions. Folia Biol..

[41] Herborn K.A., Macleod R., Miles W.T.S., Schofield A.N.B., Alexander L., Arnold K.E. (2010). Personality in captivity reflects personality in the wild. Animal Behav..

[42] Rosin M.Z., Kwieciński Z. (2011). Digestibility of prey by the White Stork (*Ciconia ciconia*) under experimental conditions. Ornis Fenn..

[43] Angelier F., Parenteau C., Trouve C., Angelier N. (2016). Does the stress response predict the ability of wild birds to adjust to short-term captivity? A study of the rock pigeon (*Columbia livia*). R. Soc. Open Sci..

[44] Kwieciński Z., Rosin Z.M., Dylewski Ł., Skórka P. (2017). Sexual differences in food preferences in the white stork: An experimental study. Sci. Nat..

[45] Kwieciński Z., Krawczyk A., Ćwiertnia P. (2009). Effect of ambient temperature on selected aspects of incubation behaviour in the Golden Eagle *Aquila chrysaetos* under aviary conditions. Ornis Pol..

[46] Schaub M., Zink R., Beissmann H., François Sarrazin F., Arlettaz R. (2009). When to end releases in reintroduction programs: Demographic rates and population viability analysis of bearded vultures in the Alps. J. App. Ecol..

[47] Hausknecht R., Jacobs S., Müller J., Zink R., Frey H., Solheim R., Vrezec A., Kristin A., Mihok J., Kergalve I. (2013). Phylogeographic analysis and genetic cluster recognition for the conservation of Ural Owls (*Strix uralensis*) in Europe. J. Ornithol..

[48] Deeming D.C., Jarrett N.S., Deeming D.C., Reynolds S.J. (2015). Applications of incubation science to aviculture and conservation. Nests, Eggs, and Incubation.

[49] Rosenberger J., Lukaszewicz E., Kowalczyk A., Deeming D.C., Rzonca Z. (2016). Nesting behaviour of Capercaillie (*Tetrao urogallus*) females kept in aviaries. Ornis Fenn..

[50] Skutch A.F. (1962). The constancy of incubation. Wilson Bull..

[51] Deeming D.C., Deeming D.C. (2002). Behavior patterns during incubation. Avian Incubation: Behaviour, Environment, and Evolution.

[52] Kirkham C.B.S., Davis S.K. (2013). Incubation and nesting behaviour of the Chestnut-collared Longspur. J. Ornithol..

[53] Deeming C.D., Deeming D.C. (2002). Patterns and significance of egg turning. Avian Incubation: Behaviour, Environment, and Evolution.

[54] Zar J.H. (1999). Biostatistical Analysis.

[55] Hałupka K. (1994). Incubation feeding in Meadow Pipit *Anhus pratensis* affects female time budget. J. Avian Biol..

[56] Albertini E. (2006). Breeding and keeping the Great Grey Owl (*Strix nebulosa*). Tyto.

[57] Kloskowski J. (1999). Prolonged incubation of unhatchable eggs in Red-necked Grebes (*Podiceps grisegena*). J. Ornithol..

[58] Marks J.S. (1983). Prolonged in Cubation by a Long-eared Owl. J. Field Ornithol..

[59] Margalida A.A., Arroya B.E., Bortolotti G.R., Bertran J. (2006). Prolonged incubation in raptors: Adaptive or nonadaptive behavior?. J. Raptor Res..

[60] Drent R., Farner D.S., King J.R. (1975). Incubation. Avian Biology Vol 5.

[61] Wuczyński A. (2012). Prolonged Incubation and Early Clutch Reduction of White Storks (*Ciconia ciconia*). Wilson J. Ornithol..

[62] Sutcliffe S. (1982). Prolonged Incubation Behavior in Common Loons. Wilson Bull..

[63] Holcomb L.C. (1970). Prolonged incubation behaviour of Red-winged Blackbird incubating several egg sizes. Behaviour.

[64] Afik D., Ward D. (1989). Incubation of Dead Eggs. Auk.

[65] Weeb D.R. (1987). Thermal tolerance of avian embryos: A review. Condor Ornithol. Appl..

[66] Bergstom P.W. (1989). Incubation temperatures of Wilson’s Plovers and Killdeers. Condor.

[67] Carey C., Rahn H., Parisi P. (1980). Calories, water, lipid and yolk in avian eggs. Condor.

[68] Boersma P.D., Wheelwright N.T. (1979). Egg neglect in the Procellariiformes: Reproductive adaptations in the Fork-tailed Storm-Petrel. Condor.

[69] Deeming D.C., Dickinson A.M., Broughton R.E., Locke E., Gray L.A., Bennett S.L., Gilchrist R., Muniz S., Goodman A.M., Biddle L.E. (2020). Factors affecting thermal insulation of songbird nests as measured using temperature loggers. Physiol. Biochem. Zool..

[70] DuRant S.E., Hopkins W.A., Hepp G.R., Walters J.R. (2013). Ecological, evolutionary, and conservation implications of incubation temperature-dependent phenotypes in birds. Biol. Rev..

[71] Haftorn S. (1988). Incubating female passerines do not let the egg temperature fall below the ‘physiological zero temperature’ during their absences from the nest. Ornis Scand..

[72] Cooper C.B., Voss M.A. (2013). Avian incubation patterns reflect temporal changes in developing clutches. PLoS ONE.

[73] Forsman D. (1980). Ageing and moult in Western Palearctic Hawk Owls *Surnia u. ulula* L.. Ornis Fenn..

[74] Payne R.B., Farner D.S., King J.R. (1972). Mechanisms and control of moults. Avian Biology Vol 2.

[75] Pyle P. (1997). Flight-Feather Moult Patterns and Age in North American Owls.

[76] Harms C.T. (2021). Incubation period behaviour of a pair of Eurasian Eagle-owls (*Bubo bubo*) based on IR-video recordings at a nest site in Baden-Württemberg, Germany in 2015. AIRO.

